# Паттерн биохимических маркеров минеральных и костных нарушений у реципиентов почечного трансплантата: опыт одного центра

**DOI:** 10.14341/probl13167

**Published:** 2023-05-11

**Authors:** А. В. Ватазин, Е. В. Паршина, Р. О. Кантария, В. А. Степанов, А. Б. Зулькарнаев

**Affiliations:** Московский областной научно-исследовательский клинический институт им. М.Ф. Владимирского; Санкт-Петербургский государственный университет; Московский областной научно-исследовательский клинический институт им. М.Ф. Владимирского; Московский областной научно-исследовательский клинический институт им. М.Ф. Владимирского; Московский областной научно-исследовательский клинический институт им. М.Ф. Владимирского

**Keywords:** трансплантация почки, гиперпаратиреоз, паратиреоидный гормон, гиперкальциемия, витамин D

## Abstract

**АКТУАЛЬНОСТЬ:**

АКТУАЛЬНОСТЬ. На сегодняшний день крайне мало исследований, представляющих объективные данные о распространенности минеральных и костных нарушений (МКН) у реципиентов почечного трансплантата (ПТ) в России. ЦЕЛЬ. Провести скрининг, включающий определение основных лабораторных показателей МКН у пациентов, перенесших аллотрансплантацию трупной почки (АТП), а также оценить назначение сопроводительной терапии, направленной на коррекцию МКН при хронической болезни почек (МКН-ХБП).

**МАТЕРИАЛЫ И МЕТОДЫ:**

МАТЕРИАЛЫ И МЕТОДЫ. В поперечное исследование были включены 236 пациентов, перенесших успешную трансплантацию почки. У всех пациентов определяли уровень интактного паратиреоидного гормона (ПТГ), общего кальция, фосфора, щелочной фосфатазы (ЩФ) сыворотки.

**РЕЗУЛЬТАТЫ:**

РЕЗУЛЬТАТЫ. Лишь у 6,2% реципиентов ПТ наблюдались целевые уровни всех исследуемых лабораторных показателей МКН, при этом повышенный уровень ПТГ в сочетании с гиперкальциемией был отмечен почти у трети пациентов (31%). Целевой уровень ПТГ наблюдался у 13% реципиентов, 84% пациентов демонстрировали гиперпаратиреоз. Доли пациентов с целевым уровнем ПТГ не различались в группах реципиентов с сохранной и сниженной расчетной скоростью клубочковой фильтрации (рСКФ) (p=0,118). Гиперкальциемия наблюдалась у 29% реципиентов. Уровни неорганического фосфора сыворотки значительно различались в группах пациентов с разной рСКФ (p<0,0001), возрастая по мере снижения функции трансплантата. У 40,7% пациентов было отмечено повышение уровня ЩФ. В качестве сопутствующей терапии 123 пациента получали активный витамин D (альфакальцидол), 33 пациента — препарат неактивной формы витамина D (колекальциферол), 57 пациентов не получали лекарственной терапии МКН-ХБП. Уровень общего кальция сыворотки статистически значимо различался в этих группах (p=0,0006), наиболее высокий его уровень отмечался в группе терапии колекальциферолом. Доля пациентов с нормокальциемией в группе лечения колекальциферолом была наиболее низкой (χ2 р=0,0018).

**ЗАКЛЮЧЕНИЕ:**

ЗАКЛЮЧЕНИЕ. Распространенность МКН у реципиентов ПТ очень высока. Применение альфакальцидола по сравнению с препаратами неактивной формы витамина D может быть более безопасным в отношении развития гиперкальциемии.

## Abstract (en)

**BACKGROUND:**

BACKGROUND: There is a lack of studies providing comprehensive data on the prevalence of mineral and bone disorders (MBD) laboratory abnormalities after kidney transplantation in Russia.

**AIM:**

AIM: to obtain real-world data on the prevalence of the main mineral abnormalities among kidney transplant recipients and to revise their concomitant MBD therapy.

**METHOD:**

METHOD: This cross-sectional study included 236 patients with successful kidney transplantation. Their serum intact parathyroid hormone (iPTH), total calcium (Ca), phosphorus (P), and alkaline phosphatase (ALP) levels were measured.

**RESULTS:**

RESULTS: Only 6.2% of our cohort had all laboratory parameters within the target range, whereas persistent HPT along with hypercalcemia was noted in almost one third of the patients (31%). Normal iPTH levels were observed in 13% cases; 84% of the patients had hyperparathyroidism. The fraction of patients with target iPTH did not differ between the groups with normal and decreased estimated glomerular filtration rate (eGFR) (p=0.118). Hypercalcemia was observed in 29% cases. The serum P level varied significantly in groups with different eGFR (p<0.0001), increasing with declining graft function. Furthermore, 40.7% of patients had ALP above the target range. While 123 patients received active vitamin D (alfacalcidol), 33 received monotherapy with inactive vitamin D (cholecalciferol). The control group consisted of 57 medication-naïve patients. The serum total Ca level varied significantly between the groups (p=0.0006), being higher in patients supplemented with cholecalciferol. The fraction of patients with normocalcemia was lowest in the cholecalciferol group (chi-square, р=0.0018).

**CONCLUSION:**

CONCLUSION: The prevalence of biochemical abnormalities after kidney transplantation is high. Alfacalcidol usage may be safer than using cholecalciferol to prevent hypercalcemia development.

## ВВЕДЕНИЕ

Количество пациентов с хронической болезнью почек (ХБП), нуждающихся в заместительной почечной терапии (ЗПТ), неуклонно растет во всех странах мира, включая Российскую Федерацию [1]. Аллотрансплантация почки (АТП) является оптимальным методом ЗПТ, обеспечивающим наилучшую выживаемость и качество жизни пациентов с ХБП [2]. Однако далеко не всегда успешное выполнение АТП приводит к полному обратному развитию минеральных и костных нарушений (МКН), распространенность которых при ХБП С3–С5Д практически универсальна. Персистирующий посттрансплантационный (третичный) гиперпаратиреоз (ТГПТ) и посттрансплантационные нарушения метаболизма костной ткани впервые были описаны еще в 70-х гг. прошлого века [3][4], спустя всего полтора десятилетия после внедрения АТП в клиническую практику. Неблагоприятные последствия этого состояния включают повышенный риск переломов, сердечно-сосудистых осложнений и общей смертности реципиентов ПТ, а также риск утраты трансплантата [5][6]. Актуальные международные и национальные клинические рекомендации предусматривают активный мониторинг показателей кальций-фосфорного обмена, минеральной плотности кости у данной группы пациентов, медикаментозную коррекцию, а при ее неэффективности — и хирургическое лечение ТГПТ [7][8]. Вместе с тем исследований, представляющих объективные данные о распространенности МКН у реципиентов почечного трансплантата (ПТ) в России, крайне мало. Руководствуясь этой целью, мы провели скрининг, включающий определение основных лабораторных показателей МКН-ХБП у когорты пациентов, перенесших АТП, а также прием сопроводительной терапии, направленной на коррекцию МКН-ХБП.

## МАТЕРИАЛЫ И МЕТОДЫ

Дизайн исследования

В поперечное ретроспективное исследование был включен 391 пациент, перенесший аллотрансплантацию трупной почки в ГБУЗ «МОНИКИ им. М.Ф. Владимирского» в период с 2007 по 2019 г. Из них в анализ были включены данные 236 пациентов (114 мужчин, 122 женщин) со сроком наблюдения после АТП более 3 мес, для которых были доступны результаты анализов крови на интактный паратиреоидный гормон (ПТГ), общий кальций, фосфор, щелочную фосфатазу (ЩФ) и креатинин, измеренные одномоментно в рамках одного визита. Проведение исследования одобрено Независимым комитетом по этике при ГБУЗ МО «МОНИКИ им. М.Ф. Владимирского» без подписания информированного согласия для участников ввиду его неинтервенционного характера (Протокол № 12 от 08.07.2021). Все процедуры были проведены в соответствии с Хельсинкской и Стамбульской декларациями.

Сбор данных

Основные клинические и демографические данные, включающие возраст, пол, лабораторные показатели, модальность и длительность ЗПТ диализом, а также данные о сопутствующей терапии, были взяты из первичной медицинской документации (истории болезни). Возраст больных колебался от 21 до 74 лет, составляя в среднем 49 [ 39; 58] лет. Всем пациентам была выполнена первичная АТП. Основываясь на данных исследования P. Evenepoel и соавт. о естественном течении МКН после АТП [9], в котором было показано, что наиболее выраженное снижение уровня ПТГ происходит в первые 3 мес после трансплантации почки, и в дальнейшем его спонтанного снижения ожидать не стоит, мы включали в исследование только реципиентов со сроком наблюдения более 3 мес после АТП. Медиана срока наблюдения после АТП составляла 42 [ 19; 75] мес, при этом у части пациентов он составлял менее года (n=33). Средняя расчетная скорость клубочковой фильтрации (рСКФ) включенных в исследование пациентов составляла 51,1±21,8 мл/мин/1,73 м2. При этом у 85 (36%) реципиентов ПТ функция трансплантата была сохранной (рСКФ более 60 мл/мин/1,73 м2). Длительность нахождения больных на ЗПТ диализом составляла 21 [ 11; 36, от 1 до 379] мес. Гемодиализ получали 155 больных (65,6 %), перитонеальный диализ — 41 больной (17,4%), у 25 (10,6%) пациентов имела место конверсия видов диализной терапии. Суммарный стаж ЗПТ (диализ + АТП) для пациентов в нашем исследовании составил 70 [ 43; 96] мес. У 15 пациентов (6,4%) АТП была проведена до начала диализа. Для оценки функции трансплантированной почки использовалась формула расчета скорости клубочковой фильтрации CKD-EPI [10]. Оценку результатов основных показателей минерального и костного обмена проводили в соответствии с целевыми диапазонами, установленными Национальными клиническими рекомендациями по МКН при хронической болезни почек [8]. Посттрансплантационный гиперпаратиреоз определяли как повышение уровня ПТГ более целевых значений для реципиентов ПТ с учетом рСКФ. Для реципиентов ПТ с сохранной (ХБП С1Т, С2Т, рСКФ >60 мл/мин/1,73 м2) или умеренно сниженной (соответствующей ХБП С3Т) функциями трансплантата целевым уровнем ПТГ считали 35–70 пг/мл, для ХБП С4Т — 70–110 пг/мл, для ХБП С5Т — 110–150 пг/мл. Также были собраны анамнестические сведения, касающиеся медикаментозной терапии МКН-ХБП после АТП.

У части пациентов были доступны данные о претрансплантационных значениях ПТГ (n=200), общего кальция (n=214), фосфора (n=222) и ЩФ (n=229) крови давностью не более 3 мес до выполнения АТП.

Статистический анализ

Для оценки количественных показателей на предмет соответствия нормальному распределению использовался модифицированный критерий Шапиро–Уилка (модификация Ройстона). Переменные, имеющие нормальное распределение, описывались как среднее ± стандартное отклонение (M±SD). Переменные, распределение которых отличалось от нормального, описывались при помощи значений медианы (Me) и нижнего и верхнего квартилей [Q1; Q3]. Для сравнения независимых совокупностей в случаях отсутствия признаков нормального распределения данных использовался U-критерий Манна–Уитни. Номинальные данные описывались с указанием абсолютных значений и процентных долей. Сравнение номинальных данных проводилось при помощи критерия χ2 Пирсона, при необходимости проведения множественных сравнений применялась процедура вычисления средней доли ложных отклонений гипотез (False Discovery Rate — FDR, метод Benjamani, Krieger and Yekutieli) [11]. Статистическая значимость различий количественных показателей, имеющих нормальное распределение, между несколькими группами оценивалась при помощи однофакторного дисперсионного анализа (ANOVA), в случае обнаружения статистически значимых различий между группами проводилось их сравнение попарно при помощи апостериорного критерия Тьюки. При сравнении количественных данных, имеющих отличное от нормального распределение, между несколькими группами использовался тест Краскела–Уоллиса, при наличии различий дополнительно проводилось попарное сравнение при помощи критерия Манна–Уитни с FDR-коррекцией уровня значимости. С целью изучения связи между явлениями, представленными количественными данными, распределение которых отличалось от нормального, использовался непараметрический метод — расчет коэффициента ранговой корреляции Спирмена. Рассчитывали коэффициент корреляции ρ, его 95% доверительный интервал и р-значение. Скрининговую информативность количественных показателей оценивали при помощи ROC-анализа. Оптимальное пороговое значение количественного признака определяли на основании максимизации отношения правдоподобия. Для выбранного порогового значения рассчитывали относительный риск (relative risk, RR) и отношение шансов (odds ratio, OR), а также 95% доверительный интервал (ДИ) этих оценок. Статистический анализ проводился с использованием программы GraphPad Prism v.9.0.0. Оценивался двусторонний уровень значимости. Значения p<0,05 считались статистически значимыми.

## РЕЗУЛЬТАТЫ

Паратиреоидный гормон

Медиана уровня интактного ПТГ реципиентов ПТ составила 120 [ 87; 189] пг/мл. Значения ПТГ, соответствующие разной функции почечного трансплантата, представлены в табл. 1. Закономерно уровни ПТГ, соответствующие разным стадиям ХБП, различались статистически значимо (р<0,0001, тест Краскела–Уоллиса). При попарном сравнении почти все группы достоверно отличались друг от друга по уровню ПТГ (рис. 1). Отмечена обратная корреляция средней силы между уровнем ПТГ и рСКФ реципиентов ПТ (ρ=-0,454 [ 95% ДИ: -0,55; -0,34], р<0,0001). Также значения ПТГ слабо коррелировали со сроком наблюдения пациентов после АТП (ρ=0,2 [ 95% ДИ: 0,07; 0,32], р=0,0025), а также с суммарной продолжительностью ЗПТ (ρ=0,17 [ 95% ДИ: 0,04; 0,3], р=0,008).

При этом доля пациентов, находящихся в целевом диапазоне ПТГ с учетом функции почечного трансплантата, составила 12,7%, ниже целевого — 3%, выше целевого — 84,3%. Доли пациентов с различными уровнями ПТГ статистически значимо различались при сравнении групп реципиентов со сниженной (менее 60 мл/мин/1,73 м2) и нормальной функцией трансплантата (рис. 2А). Однако при сравнении долей пациентов в целевом и отличном от целевого диапазонах ПТГ в группах с сохранной и сниженной рСКФ достоверных различий установлено не было (рис. 2В).

У 14 пациентов на момент скрининга уровень ПТГ находился в диапазоне от 300 до 600 пг/мл, у 7 пациентов — превышал 600 пг/мл.

Медиана уровня ПТГ до АТП была несколько выше у пациентов с посттрансплантационным ГПТ по сравнению с реципиентами, имевшими нормальный уровень ПТГ: 346 [ 213; 595] и 250 [ 100; 522] пг/мл соответственно, р=0,0105. Риск развития посттрансплантационного ГПТ был выше у пациентов с исходным уровнем ПТГ более 157 пг/мл: RR=1,4 [ 95% ДИ: 1,1; 1,96], р=0,0002. Мы не обнаружили связи посттрансплантационного ГПТ со стажем диализа (p=0,57), сроком АТП (р=0,13), претрансплантационными уровнями общего кальция (р=0,33), фосфора (р=0,06) и ЩФ сыворотки (р=0,75).

Общий кальций

Медиана значений общего кальция сыворотки составила 2,41 [ 2,36; 2,56] ммоль/л. У 68% реципиентов ПТ уровень кальция находился в пределах целевых значений (2,1–2,5 ммоль/л), ниже целевого диапазона — у 3%, выше — у 29%. Уровни общего кальция для пациентов с различной функцией ПТ, представленные в табл. 1, различались статистически значимо (р=0,04, тест Краскела-Уоллиса), однако после коррекции уровня значимости достоверных различий при сопоставлении групп попарно выявлено не было. Также не было выявлено корреляции уровней общего кальция с рСКФ (р=0,132), сроком наблюдения после АТП (р=0,06). Вместе с тем значения общего кальция слабо коррелировали с суммарной продолжительностью ЗПТ (ρ=0,2 [ 95% ДИ: 0,07; 0,32], р=0,002). Также наблюдалась слабая положительная корреляция уровня кальция с уровнем ПТГ сыворотки (ρ=0,282 [ 95% ДИ: 0,15; 0,4], р<0,0001), а также с уровнем ЩФ (ρ=0,181 [ 95% ДИ: 0,05; 0,31], р=0,006).

В течение первых 12 мес после АТП гиперкальциемия наблюдалась у 21% (7/33) пациентов, после года после проведенной АТП — у 30% (61/203) пациентов.

Медиана диализного стажа в группе реципиентов с гиперкальциемией была несколько выше по сравнению с пациентами, демонстрировавшими нормокальциемию: 24 [ 12; 40] и 14 [ 8; 34] мес соответственно, р=0,013. Пациенты с более продолжительным стажем диализа до АТП имели более высокий риск развития гиперкальциемии после АТП, при этом риск в наибольшей степени возрастал для пациентов со стажем диализа более 19 мес: RR=1,8 [ 95% ДИ: 1,2; 2,7], OR=2,2 [ 95% ДИ: 1,2; 4], р=0,0066.

Медианы претрансплантационных значений ПТГ были выше в группе пациентов с гиперкальциемией по сравнению с пациентами с нормокальциемией: 407 [ 289; 736] против 300 [ 190; 496] пг/мл соответственно, р=0,0004. Пациенты с повышенным уровнем ПТГ до АТП имеют существенно больший риск гиперкальциемии по отношению к пациентам с нормальным ПТГ, при этом в наибольшей степени риск возрастает для пациентов с ПТГ более 600 пг/мл (RR=2,1 [ 95% ДИ: 1,35; 3,3], OR=3,1 [ 95% ДИ: 1,6; 6,2], р=0,0015). Пациенты с уровнем ПТГ до АТП более 300 пг/мл также имели существенно более высокий риск гиперкальциемии после АТП по сравнению с пациентами, чей уровень ПТГ составлял менее 300 пг/мл: RR=1,85 [ 95% ДИ: 1,14; 3], OR=2,3 [ 95% ДИ: 1,2; 4,4], р=0,012.

Медиана претрансплантационных значений общего кальция в группе реципиентов с гиперкальциемией была выше по сравнению с пациентами, демонстрировавшими нормокальциемию: 2,4 [ 2,26; 2,54] и 2,31 [ 2,2; 2,42] ммоль/л соответственно, р=0,003. Пациенты с более высоким уровнем кальция до АТП имели более высокий риск развития гиперкальциемии после АТП, при этом риск в наибольшей степени возрастал для пациентов с уровнем общего кальция более 2,39 ммоль/л: RR=1,9 [ 95% ДИ: 1,3; 2,9], OR=2,5 [ 95% ДИ: 1,4; 4,5], р=0,002.

Мы не обнаружили связи посттрансплантационной гиперкальциемии с возрастом (р=0,69), полом реципиентов (р=0,72), стажем АТП (р=0,4), фактом терапии МКН-ХБП до АТП (p=0,64).

Фосфор

Медиана уровня фосфора сыворотки составила 1,08 [ 1,0; 1,26] ммоль/л. Доля пациентов в целевом диапазоне, согласно Национальным рекомендациям (0,87–1,49 ммоль/л), составила 79,7%, ниже целевого уровня — 9,7%, выше — 10,6%. При этом в подгруппе пациентов, срок наблюдения которых после проведенной АТП не превышал 12 мес, доля пациентов с гипофосфатемией была существенно выше по сравнению с пациентами, которым АТП была выполнена за год и ранее до скрининга: 30,3% (10/33) пациентов против 6,4% (13/203) пациентов соответственно (р=0,0002).

Значения фосфатемии, соответствующие различным стадиям ХБП у реципиентов ПТ, представлены в табл. 1. Ожидаемо различия уровня фосфора сыворотки в группах с разной рСКФ статистически значимо различались (р<0,0001, тест Краскела-Уоллиса). Наличие достоверных различий при попарном сравнении групп после коррекции уровня значимости представлено на рис. 3. Отмечена слабая обратная корреляция между значениями фосфора сыворотки и рСКФ пациентов (ρ=-0,137 [ 95% ДИ: -0,26; -0,005], р=0,036). Корреляции между уровнем фосфатемии и сроком наблюдения после АТП, суммарной продолжительностью ЗПТ обнаружено не было (р=0,42 и р=0,54 соответственно).

Щелочная фосфатаза

Медиана значений ЩФ в нашей выборке составила 100 [ 81; 130, от 25 до 377] Ед/л. Достоверных различий уровней ЩФ в группах реципиентов ПТ с различной функцией трансплантата выявлено не было (р=0,54, тест Краскела–Уоллиса), значения показателей представлены в табл. 1. Доля пациентов с уровнем ЩФ в целевом диапазоне, согласно Национальным рекомендациям, с учетом пола (53–128 Ед/л для мужчин, 42–98 Ед/л для женщин) составила 54,3%, ниже целевого уровня — 5%, выше — 40,7%. Уровень ЩФ слабо коррелировал с уровнем ПТГ: ρ=0,159 [ 95% ДИ 0,03; 0,28], р=0,014. Корреляции значений ЩФ с длительностью наблюдения после АТП, с общей продолжительностью ЗПТ не выявлено (р=0,07 и р=0,25 соответственно).

В целом на момент скрининга лишь 14 (6,2%) пациентов в нашей выборке демонстрировали уровни всех исследуемых показателей МКН-ХБП в пределах целевого диапазона (Рис. 4А). Сочетание повышенного уровня ПТГ и гиперкальциемии наблюдалось у 24% (20 из 85) пациентов с сохранной функцией трансплантата (Рис. 4D) и у 27% (41 из 151) пациентов со сниженной функцией трансплантата (Рис. 4С). Посттрансплантационный гиперпаратиреоз в сочетании с гиперкальциемией и гипофосфатемией (типичная триада проявлений МКН у реципиентов почечного трансплантата) наблюдался лишь у 3% пациентов в нашей выборке, в то время как сочетание повышенного ПТГ и гиперкальциемии было отмечено почти у трети пациентов (Рис. 4B).

Терапия МКН-ХБП

Данные о сопутствующей терапии на момент скрининга были доступны у 230 пациентов из 236. Терапию МКН-ХБП получали 173 (75%) реципиентов ПТ. При этом большинство пациентов (75,7%, 131/173) получали терапию препаратами активной формы витамина D (альфакальцидола), у 19,7% (34/173) пациентов применялись препараты неактивной формы витамина D (колекальциферола), 7% (12/173) пациентов получали лечение кальцимиметиками (цинакальцетом). Лечение фосфатсвязывающими препаратами получали 3 пациента из 173, антирезорбтивную терапию (деносумаб) — 2 пациента.

Нами было проведено сравнение лабораторных данных пациентов, получавших и не получавших терапию МКН-ХБП препаратами аналогов витамина D. Из анализа были исключены пациенты, принимающие препараты других групп. Суммарно лечение альфакальцидолом или колекальциферолом получали 156 пациентов, без терапии — 57 пациентов. При этом не было выявлено различий между уровнями общего кальция, ПТГ, фосфора и ЩФ при сравнении как абсолютных значений (р=0,984, р=0,07, р=0,335 и р=0,153 соответственно), так и соотношения долей пациентов в целевом диапазоне и выше него в этих двух группах (рис. 5).

При сравнении показателей в группах пациентов, получавших монотерапию альфакальцидолом (n=123), колекальциферолом (n=33) и не получавших терапии аналогами витамина D (n=57), были выявлены существенные различия уровней общего кальция (р=0,0006, тест Краскела–Уоллиса): в группе пациентов, принимавших колекальциферол, уровень кальция крови был выше по сравнению с получавшими альфакальцидол и не получавшими терапии пациентами (рис. 6). Не было выявлено различий по уровню ПТГ (р=0,171), фосфора (р=0,563), ЩФ (р=0,098) в этих трех группах.

Доля пациентов, находящихся в целевом диапазоне по уровню общего кальция сыворотки, была ниже среди тех, кто получал терапию колекальциферолом, по сравнению с получавшими альфакальцидол и не получавшими терапии реципиентами ПТ — рис. 7. Также отмечались различия в уровне ЩФ в трех группах (критерий χ2, р=0,005): доля пациентов с ЩФ выше целевого диапазона была больше среди принимавших альфакальцидол пациентов по сравнению с получающими неактивный витамин D и не получающими терапии больными (рис. 7). Различий в распределении пациентов по категориям «в пределах целевых значений» и «выше целевых значений» по уровню ПТГ и фосфора выявлено не было.

При этом в группе получавших терапию колекальциферолом средняя рСКФ была ниже — 40,5±15,9 мл/мин/1,73 м2 по сравнению с реципиентами ПТ, получавшими альфакальцидол (52,2±21,9 мл/мин/1,73 м2) и не получавшими терапии (59±20,8 мл/мин/1,73 м2) — ANOVA р=0,0003. Результаты попарных сравнений представлены на рис. 8.

**Figure:**
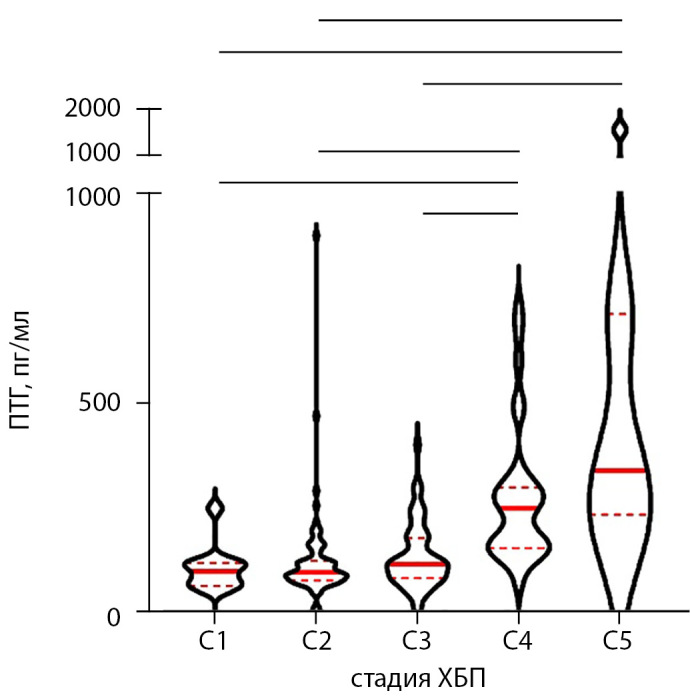
Рисунок 1. Уровни ПТГ в группах реципиентов почки с различной функцией трансплантата. Тест Краскела–Уоллиса, р<0,0001. Горизонтальными линиями указаны сравнения пар групп, для которых получены достоверные различия (скорректированные значения p-value <0,0001 во всех случаях). Приведены медианы, первый и третий квартили, форма фигур отражает распределение признака. ПТГ —паратиреоидный гормон, ХБП — хроническая болезнь почек.

**Figure:**
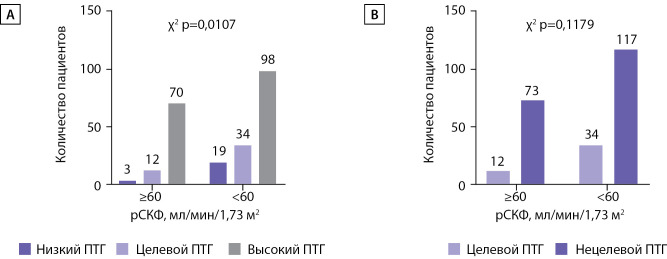
Рисунок 2. Распределение пациентов с сохранной и сниженной функцией ПТ по категориям по уровню ПТГ. ПТГ — паратиреоидный гормон, рСКФ — расчетная скорость клубочковой фильтрации.

**Table:** Таблица 1. Уровни ПТГ, общего кальция, фосфора и ЩФ в группах реципиентов ПТ с различной функцией трансплантата Примечание. ХБП — хроническая болезнь почек; ПТГ — паратиреоидный гормон; Р — фосфор сыворотки; ЩФ — щелочная фосфатаза сыворотки.

Стадия ХБП	Количество пациентов	ПТГ, пг/мл	Кальций общий,ммоль/л	Р, ммоль/л	ЩФ, Ед/л
С1 T	11	100 [ 65; 120]	2,4 [ 2,22; 2,4]	1,1 [ 1,0; 1,2]	120 [ 78; 156]
С2 T	74	98 [ 78; 124]	2,48 [ 2,4; 2,55]	1,09 [ 0,98; 1,2]	100 [ 83; 121]
С3 T	111	117 [ 83,8; 178,5]	2,4 [ 2,34; 2,54]	1,03 [ 0,91; 1,2]	100 [ 80; 130]
С4 T	31	250 [ 155; 300]	2,5 [ 2,4; 2,62]	1,15 [ 1,0; 1,44]	110 [ 94; 143]
С5 T	9	340 [ 235; 713]	2,28 [ 2,15; 2,6]	1,9 [ 1,35; 2,75]	130 [ 65,5; 210]

**Figure:**
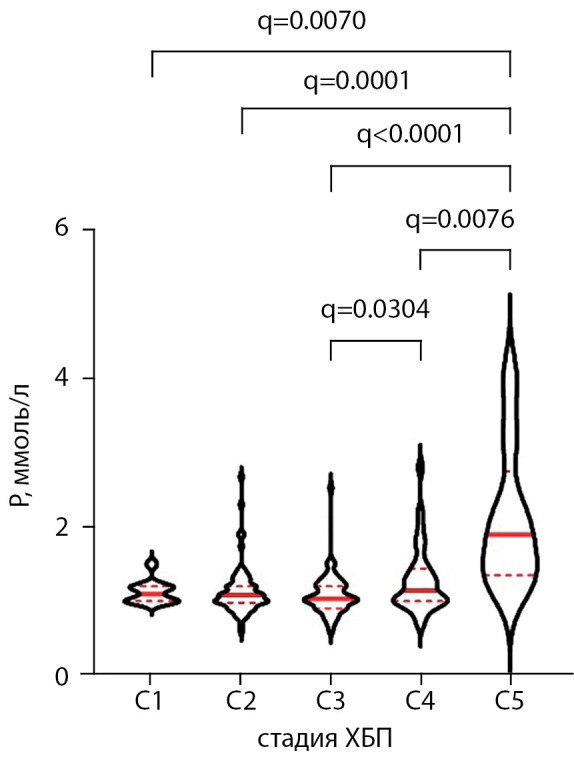
Рисунок 3. Уровни сывороточного фосфора в группах реципиентов почки с различной функцией трансплантата. Тест Краскела–Уоллиса, р<0,0001. Указаны скорректированные значения p-value (q) для достоверных различий между группами. Приведены медианы, первый и третий квартили, форма фигур отражает распределение признака. Р — фосфор сыворотки, ХБП — хроническая болезнь почек.

**Figure:**
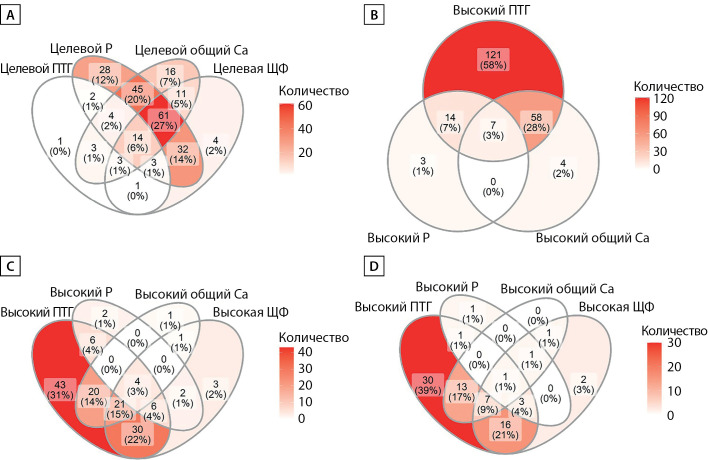
Рисунок 4. А — распространенность целевых значений ПТГ, общего кальция, фосфора и ЩФ у реципиентов почечного трансплантата. B — частота встречаемости специфических биохимических нарушений у реципиентов ПТ. С — частота встречаемости повышенных уровней ПТГ, общего кальция, фосфора и ЩФ у реципиентов ПТ с сохранной функцией трансплантата. D — частота встречаемости повышенных уровней ПТГ, общего кальция, фосфора и ЩФ у реципиентов ПТ со сниженной функцией трансплантата. ПТГ — паратиреоидный гормон, Р — фосфор сыворотки, ЩФ — щелочная фосфатаза сыворотки.

**Figure:**
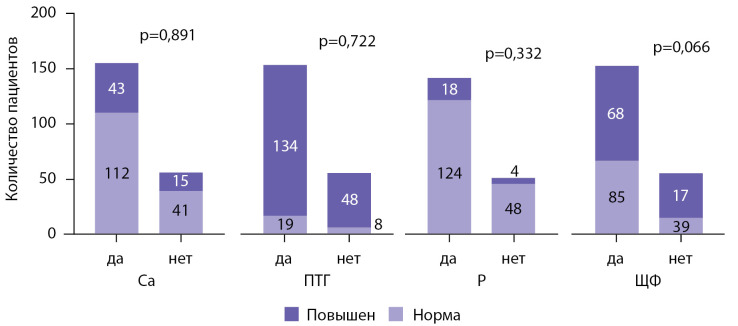
Рисунок 5. Распределение пациентов, получающих и не получающих терапию МКН-ХБП, по категориям по уровню общего кальция, ПТГ, фосфора и ЩФ. Cа — общий кальций, ПТГ — паратиреоидный гормон, Р — фосфор сыворотки, ЩФ — щелочная фосфатаза сыворотки.

**Figure:**
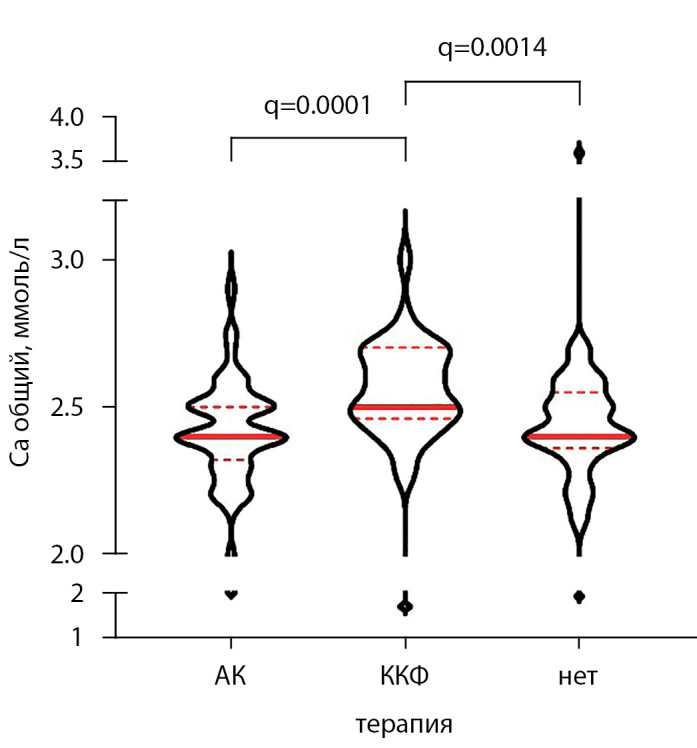
Рисунок 6. Уровни общего кальция сыворотки реципиентов ПТ в группах, получающих и не получающих терапию МКН-ХБП. АК —альфакальцидол, ККФ — колекальциферол. Тест Краскела-Уоллиса, р=0,0006. Указаны скорректированные значения p-value (q) для достоверных различий между группами. Приведены медианы, первый и третий квартили, форма фигур отражает распределение признака. АК — альфакальцидол, ККФ – колекальциферол.

**Figure:**
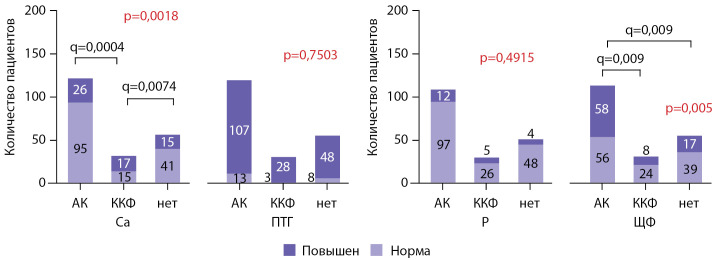
Рисунок 7. Распределение пациентов, получающих и не получающих терапию различными препаратами витамина Д, по категориям по уровню общего кальция, ПТГ, фосфора и ЩФ. Красным цветом обозначены результаты омнибусного теста, также приведены скорректированные значения p-value (q) для достоверных различий между группами. АК — альфакальцидол, ККФ — колекальциферол, Са — общий кальций, ПТГ — паратиреоидный гормон, Р — фосфор сыворотки, ЩФ — щелочная фосфатаза сыворотки.

**Figure:**
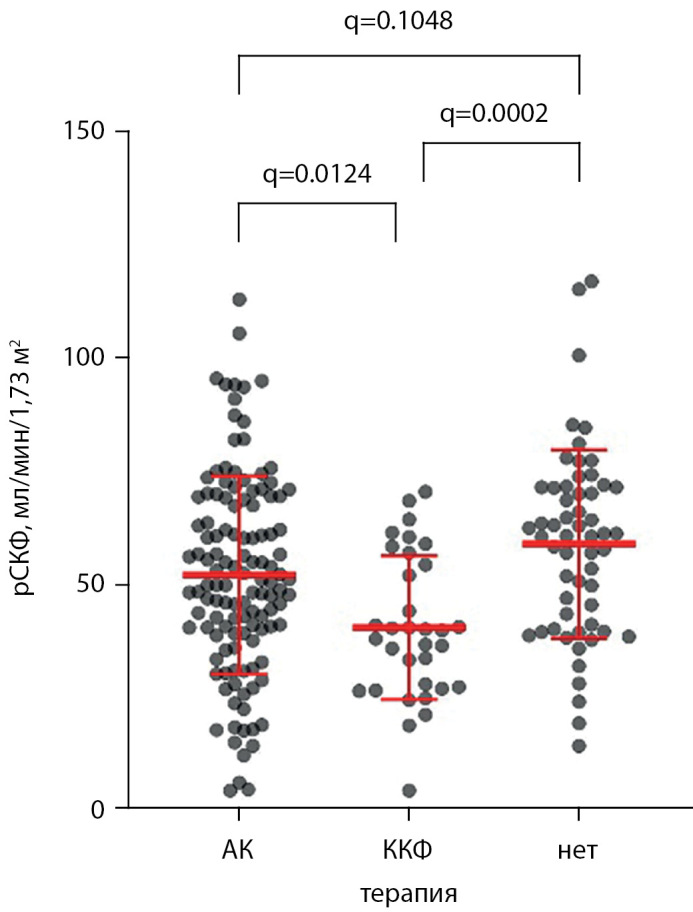
Рисунок 8. Значения рСКФ реципиентов ПТ, получающих и не получающих терапию различными препаратами витамина Д. АК — альфакальцидол, ККФ — колекальциферол. ANOVA р=0,0003. Приведены скорректированные значения p-value (q). Приведены распределение, среднее значение, стандартное отклонение среднего. АК — альфакальцидол, ККФ — колекальциферол, рСКФ — расчетная скорость клубочковой фильтрации.

## ОБСУЖДЕНИЕ

Данное исследование представляет результаты наиболее масштабного на сегодняшний день в РФ скрининга, проведенного среди реципиентов почечного трансплантата с целью оценки распространенности МКН.

Несмотря на всю привлекательность тезиса о том, что трансплантация почки является «идеальной терапией» вторичного гиперпаратиреоза, это утверждение нельзя назвать справедливым. Характерные нарушения МКН-ХБП у реципиентов ПТ включают биохимическую триаду: персистирующее повышение ПТГ, гиперкальциемию и гипофосфатемию. Хорошо известно, что персистенция гиперпаратиреоза после АТП — ТГПТ — является фактором риска сердечно-сосудистой заболеваемости, утраты трансплантата, а также общей смертности даже у пациентов с стабильной и сохранной функцией трансплантата [5, 6]. Также ТГПТ рассматривают как предиктор развития посттрансплантационной болезни кости [12, 13]. В патогенезе ТГПТ важную роль играет предшествующая гиперплазия околощитовидных желез, которая относительно медленно подвергается обратному развитию; помимо этого, гиперпаратиреоз может развиваться и de novo при сниженной (субоптимальной) функции почечного трансплантата аналогично тому, как это происходит на додиализных стадиях ХБП [14].

Распространенность ТГПТ, по данным различных исследований, варьирует в широких пределах. В хорошо известном исследовании P. Evenepoel и соавт., анализирующем естественное течение МКН-ХБП у 861 реципиента ПТ, доля пациентов с повышенным уровнем ПТГ спустя 1–4 года после АТП составила 17% [9]. В другом исследовании, включавшем 360 реципиентов АТП с сохранной функцией ПТ, доля пациентов с повышенным выше референсных значений ПТГ достигала 52% [15]. В нашем исследовании персистенция ГПТ оказалась существенно более распространенной (84%), что, вероятно, отчасти может быть объяснено критериями оценки: так, в исследовании P. Evenepoel ГПТ определялся как уровень ПТГ более 100 пг/мл спустя год после АТП для всех пациентов без учета функции трансплантата. Также бóльшая доля пациентов с ПТГ выше целевого уровня в нашей выборке по сравнению с результатами приведенных выше исследований может объясняться неравной исходной распространенностью и/или тяжестью вторичного ГПТ на претрансплантационном этапе, что косвенно подтверждается результатами проведенного недавно масштабного скрининга диализных пациентов Северо-Западного федерального округа [16]. Разница в распространенности персистирующего ГПТ могла бы определяться разной продолжительностью диализного лечения до АТП, т.е. длительностью течения выраженных нарушений МКН-ХБП, однако она оказалась сопоставима у пациентов в нашем исследовании и в упомянутых работах зарубежных авторов [9][15].

Парадоксальным образом, доля пациентов с повышенным уровнем ПТГ среди пациентов с сохранной функцией трансплантата была несколько выше, чем среди пациентов с рСКФ менее 60 мл/мин/1,73 м2 (82 и 65% соответственно). Это может быть связано с определением целевого диапазона для каждой стадии ХБП, принятым в существующих клинических рекомендациях [8]. При этом доля пациентов, находящихся в целевом диапазоне значений ПТГ, не зависела от функции ПТ, что отмечено и другими авторами [9]. Стоит также отметить, что выраженное повышение уровня ПТГ (выше 300 пг/л, что соответствует превышению верхней границы нормы примерно в 2–2,5 раза) отмечалось только у 9% пациентов — вероятно, для некоторых из них в перспективе стоит рассматривать оперативное лечение гиперпаратиреоза (паратиреоидэктомию).

Будучи следствием персистирующего гиперпаратиреоза, высокообменная болезнь костной ткани по результатам биопсии наблюдается почти у половины реципиентов ПТ [17]. В нашем исследовании уровень ЩФ, косвенно отражающий интенсивность обмена в кости, был повышен у 40% скринированных пациентов, коррелируя с уровнем ПТГ сыворотки, что вполне согласуется с известными представлениями о нарушениях костного метаболизма после АТП.

Гипофосфатемия после АТП традиционно считается распространенной, достигая по оценкам некоторых исследователей 90% в раннем посттрансплантационном периоде — до 3 мес после выполнения АТП [18]. В период с 3 до 12 мес после АТП уровень фосфора у большинства пациентов возвращается к нормальным значениям [19]. P. Evenepoel и соавт. в уже упомянутом нами ранее исследовании приводят более скромные цифры: до 40% пациентов демонстрируют гипофосфатемию в период до года и 12–15% пациентов — в период после года после трансплантации почки [9]. Схожие результаты получены и нами. Развитие гипофосфатемии связывают с сохраняющимися после АТП повышенными уровнями фактора роста фибробластов (ФРФ-23) и ПТГ. Несмотря на снижение обоих этих показателей после успешной АТП, их уровень превышает таковой у пациентов с соответствующей стадией ХБП [19]. Еще одним важным фактором развития гипофосфатемии является терапия глюкокортикоидами. Так, в экспериментальных исследованиях показано, что глюкокортикоиды снижают экспрессию и активность Na-Pi-котранспортера, локализованного на поверхности клеток тубулярного эпителия проксимальных канальцев, что приводит к сниженной его абсорбции [20][21]. Существуют свидетельства в пользу того, что применение ряда других иммуносупрессивных препаратов также может вносить вклад в развитие гипофосфатемии [22–25]. Индуцированная ФРФ-23 фосфатурия вызывает деминерализацию костей, что в сочетании с кортикостероидной терапией неизбежно приводит к развитию остеопороза и, как следствие, высокой частоте переломов среди реципиентов АТП — почти в четыре раза выше, чем в общей популяции [26–28]. Вместе с тем существующие данные о влиянии гипофосфатемии на общую смертность реципиентов и риск утраты трансплантата противоречивы [18][29][30]. По мере снижения функции трансплантата сохраняющиеся повышенные уровни ФРФ-233 и ПТГ приводят к гиперфосфатемии аналогично тому, как это происходит при ХБП С3–5 до начала ЗПТ. Это хорошо прослеживается и у обследованных нами пациентов (рис. 5). В отличие от гипофосфатемии, гиперфосфатемия у реципиентов ПТ имеет четко доказанную связь как с общей смертностью, так и с риском утраты трансплантата [31][32]. При этом возможности терапии гиперфосфатемии существенно ограничены: отсутствуют данные об эффективности и безопасности фосфатсвязывающих препаратов у реципиентов ПТ, при этом известно о снижении пиковой концентрации микофенолата мофетила на фоне приема фосфат-биндеров ФСП [33].

Аналогично результатам многочисленных исследований, мы наблюдали высокую частоту гиперкальциемии у реципиентов ПТ как в течение первого года после АТП, так и в более поздние сроки после ее проведения, не связанную с функцией трансплантата на момент скрининга. Вопреки данным, указывающим на снижение доли пациентов с гиперкальциемией в динамике в отдаленном периоде после АТП [9][34], в нашей выборке она оставалась достаточно высокой, что, по всей видимости, связано с большей частотой встречаемости повышенного уровня ПТГ. Стоит отметить, что большинство обследованных нами пациентов получали терапию препаратами витамина D, что также могло повлиять на этот результат.

Поскольку наиболее существенное снижение ПТГ происходит в течение 0–3 мес после АТП, и в дальнейшем спонтанного улучшения ожидать не стоит, о начале терапии ТГПТ следует задуматься уже спустя 3 мес после выполнения успешной АТП. Актуальные клинические рекомендации KDIGO 2017 г. предлагают начинать лечение в течение первого года после трансплантации с целью нормализации биохимических показателей и предотвращения снижения минеральной плотности кости в дальнейшем; при этом отсутствуют указания на какие-либо конкретные значения ПТГ [7]. На практике чаще всего ориентируются на превышение верхней границы нормы этого показателя в 2–2,5 раза [35]. В нашей выборке в терапии МКН-ХБП нуждались три четверти всех обследованных пациентов, что лишний раз свидетельствует в пользу того, что одного лишь факта выполнения трансплантации почки недостаточно, чтобы нормализовать показатели кальций-фосфорного обмена.

Основой терапии МКН-ХБП в посттрансплантационном периоде являются препараты витамина D, поскольку снижение уровня 25(ОН) витамина D часто наблюдается у реципиентов ПТ и ассоциировано с повышением риска смерти от всех причин и риска утраты функции трансплантата [36]. В настоящее время целевые уровни 25(ОН) витамина D у реципиентов ПТ не определены, и рекомендациями KDIGO 2017 предлагается стратегия выявления и коррекции дефицита витамина D, аналогичная таковой в общей популяции. Применение неактивной формы витамина D позволяет нормализовать уровни ПТГ и кальция, однако остается неясным, влияет ли его применение на минеральную плотность кости у реципиентов ПТ [37]. Применение кальцитриола приводит к снижению уровня ПТГ и повышению минеральной плотности кости после трансплантации почки, предотвращая потерю костной массы [38]. Вместе с тем применение аналогов витамина D зачастую ограничено развитием гиперкальциемии, которая может усугублять сосудистую кальцификацию и приводить к ухудшению функции трансплантата. В нашем исследовании у половины пациентов, получавших препарат неактивной формы витамина D, было отмечено повышение уровня кальция сыворотки, в то время как применение альфакальцидола было ассоциировано с существенно меньшим риском развития гиперкальциемии. Одним из возможных объяснений может служить тот факт, что средняя рСКФ в группе принимающих колекальциферол ККФ пациентов оказалась ниже по сравнению с принимавшими альфакальцидол (40,5±15,9 и 52,2±21,9 мл/мин/1,73 м2 соответственно). Однако нельзя не признать, что различия могут быть связаны в большей степени с дозами принимаемых препаратов, различиями в показаниях к их назначению, что затруднительно оценить в рамках поперечного исследования.

К ограничениям настоящего исследования следует в первую очередь отнести его срезовый характер и опыт одного центра. Также мы не располагали анамнестическими сведениями о сопутствующей иммуносупрессивной терапии, о выполнении паратиреоидэктомии пациентам в нашей выборке. Также в исследуемой выборке не определяли уровень витамина D, отсутствовала возможность коррекции уровня общего кальция на альбумин, что могло повлиять на результаты. Наблюдательный характер исследования и отсутствие данных о дозировках и возможных изменениях лекарственной терапии не позволяют однозначно судить о наличии причинно-следственной связи между приемом препаратов и уровнем кальция, что диктует необходимость проведения проспективных исследований, направленных на изучение этого вопроса. В то же время сильной стороной исследования можно считать факт сплошного включения всех реципиентов ПТ с различным уровнем функции трансплантата, что отражает реальную клиническую практику, а также определение целевых показателей МКН-ХБП с учетом функции трансплантата.

## ЗАКЛЮЧЕНИЕ

По результатам проведенного скрининга выявлена высокая частота встречаемости нарушений основных показателей кальций-фосфорного обмена у реципиентов ПТ на различных сроках наблюдения после АТП. Наиболее часто встречались следующие биохимические отклонения: повышенный уровень ПТГ (84% пациентов), общего кальция (29%), ЩФ сыворотки (40,7%). Гипофосфатемия наблюдалась примерно у трети пациентов в первые 12 мес после АТП, в дальнейшем доля ее существенно снижалась. Уровни ПТГ и фосфатов сыворотки, но не общего кальция и ЩФ, имели достоверную связь с функцией трансплантата на момент обследования. Риск посттрансплантационного ГПТ был выше у пациентов с более высокими исходными значениями ПТГ до АТП. Риск посттрансплантационной гиперкальциемии был выше у пациентов с более высокими исходными значениями ПТГ и общего кальция до АТП, а также имевших более продолжительный стаж диализа. В целом лишь у 6% реципиентов ПТ наблюдались целевые уровни всех исследуемых лабораторных показателей МКН, при этом повышенный уровень ПТГ в сочетании с гиперкальциемией был отмечен почти у трети пациентов. Полученные нами данные свидетельствуют в пользу того, что применение препаратов активной формы витамина D у реципиентов ПТ может быть ассоциировано с меньшим риском развития гиперкальциемии, что требует проведения проспективных исследований для подтверждения.

## ДОПОЛНИТЕЛЬНАЯ ИНФОРМАЦИЯ

Источники финансирования. Работа выполнена по инициативе авторов без привлечения финансирования.

Конфликт интересов. Авторы декларируют отсутствие явных и потенциальных конфликтов интересов, связанных с содержанием настоящей статьи.

Участие авторов. Все авторы одобрили финальную версию статьи перед публикацией, выразили согласие нести ответственность за все аспекты работы, подразумевающую надлежащее изучение и решение вопросов, связанных с точностью или добросовестностью любой части работы.
